# Erector Spinae Plane Block versus Paravertebral Block after Thoracic Surgery for Lung Cancer: A Propensity Score Study

**DOI:** 10.3390/cancers15082306

**Published:** 2023-04-14

**Authors:** Benjamin Durey, Zoubir Djerada, Fairuz Boujibar, Emmanuel Besnier, François Montagne, Jean-Marc Baste, Marie-Mélody Dusseaux, Vincent Compere, Thomas Clavier, Jean Selim

**Affiliations:** 1 Department of Anaesthesiology and Critical Care, CHU Rouen, 76000 Rouen, France; b.durey@hopital-foch.com (B.D.); emmanuel.besnier@chu-rouen.fr (E.B.); marie-melody.dusseaux@chu-rouen.fr (M.-M.D.); vincent.compere@chu-rouen.fr (V.C.); thomas.clavier@chu-rouen.fr (T.C.); 2 Department of Medical Pharmacology, University of Reims Champagne-Ardenne, EA3801, SFR CAP-Santé, 51000 Reims, France; zoubir.djerada@univ-reims.fr; 3 Univ Rouen Normandie, INSERM EnVI UMR 1096, 76000 Rouen, France; fairuz.boujibar@chu-rouen.fr (F.B.); jean-marc.baste@chu-rouen.fr (J.-M.B.); 4 Department of Thoracic Surgery, Rouen University Hospital, 76000 Rouen, France; montagne-f@ch-valenciennes.fr

**Keywords:** lung cancer, erector spinae plane block, paravertebral block, post-operative pain, analgesia, video-assisted thoracic surgery

## Abstract

**Simple Summary:**

One of the major issues in thoracic surgery is adequate post-operative pain management. Inadequate management leads to complications, such as atelectasis, pneumonia, respiratory failure, and the development of chronic pain. Paravertebral block (PVB) has now become the first-line locoregional technique for video-assisted thoracic surgery (VATS) or robot-assisted thoracic surgery (RATS). New chest wall blocks, such as the erector spinae plane block (ESPB) have been described in recent years to facilitate analgesia and limit the risk of puncturing adjacent structures (neuroaxial, pleural, or vascular punctures). The ESPB is more superficial than the PVB and some studies suggest that the anesthetic could spread into the thoracic paravertebral space. Therefore, the ESPB may have fewer complications and be more effective than PVB. We hypothesize that the ESPB could decrease post-operative pain compared to PVB block after VATS or RATS for lung cancer.

**Abstract:**

Introduction: The prevention of respiratory complications is a major issue after thoracic surgery for lung cancer, and requires adequate post-operative pain management. The erector spinae plane block (ESPB) may decrease post-operative pain. The objective of this study was to evaluate the impact of ESPB on pain after video or robot-assisted thoracic surgery (VATS or RATS). Methods: The main outcome of this retrospective study with a propensity score analysis (PSA) was to compare the post-operative pain at 24 h at rest and at cough between a group that received ESPB and a group that received paravertebral block (PVB). Post-operative morphine consumption at 24 h and complications were also assessed. Results: One hundred and seven patients were included: 54 in the ESPB group and 53 in the PVB group. The post-operative median pain score at rest and cough was lower in the ESPB group compared to the PVB group at 24 h (respectively, at rest 2 [1; 3.5] vs. 2 [0; 4], *p* = 0.0181, with PSA; ESPB −0.80 [−1.50; −0.10], *p* = 0.0255, and at cough (4 [3; 6] vs. 5 [4; 6], *p* = 0.0261, with PSA; ESPB -1.48 [−2.65; −0.31], *p* = 0.0135). There were no differences between groups concerning post-operative morphine consumption at 24 h and respiratory complications. Conclusions: Our results suggest that ESPB is associated with less post-operative pain at 24 h than PVB after VATS or RATS for lung cancer. Furthermore, ESPB is an acceptable and safe alternative compared to PVB.

## 1. Introduction

One of the major issues in thoracic surgery is adequate post-operative pain management. Inadequate management leads to complications, such as atelectasis, pneumonia, respiratory failure, and the development of chronic pain [1]. In lung resection surgery, post-operative multimodal analgesia with the use of locoregional anesthesia and the improvement of the surgical techniques (video-assisted thoracic surgery (VATS) or robot-assisted thoracic surgery (RATS)) have contributed to reduced complications compared to classical thoracotomy [2]. Concerning the management of post-operative pain in thoracic surgery, epidural anesthesia (EA) has been the gold standard for a long time, but a recent meta-analysis showed that paravertebral block (PVB) was not inferior to EA and had fewer side effects [3]. PVB has now become the first-line locoregional technique for VATS or RATS. New chest wall blocks, such as the erector spinae plane block (ESPB), have been described in recent years to facilitate analgesia and limit the risk of puncturing adjacent structures. Since this first description in 2016 by Forero, a growing literature suggested it as an alternative to the PVB in many indications [4]. The ESPB is part of the interfacial plane block. In ESPB, the large volume of local anesthesia injected between the planes of the thoracolumbar fascial planes under the erector spinae muscle may impact several intercostal nerves. Opposing views on the diffusion of the drug have been reported, with no clear consensus on the mechanism of action. Some studies suggested that the anesthetic spreads into the thoracic paravertebral space [5,6], while other studies indicated that it extends to the outer surface of the chest wall, but not into the thoracic paravertebral space [7].

However, the studies performed are heterogeneous and the randomized studies conducted are non-inferiority studies with a limited number of patients [8,9,10]. Thus, the objective of this propensity score study was to evaluate the post-operative pain after thoracic surgery between a group having PVB (PVB group) and a group having ESPB (ESPB group) after VATS or RATS.

## 2. Materials and Methods

### 2.1. Population Selection

We conducted an observational retrospective study in a tertiary care hospital in Rouen, France. Patients undergoing VATS or RATS for lobectomy or segmentectomy with ESPB or PVB between May 2020 and May 2021 were included. ESPB or PVB was provided by 7 different anesthesiologists and was left to the appreciation of the anesthesiologist based on the locoregional anesthesia risks and patients’ comorbidities. The study was approved by the Ethics and Evaluation Committee for Non-Interventional Research of Rouen University Hospital (CERNI E2021-87). According to the current French law, this retrospective study dispensed with the requirement for written informed consent for each patient [11]. The list of procedures using the French nomenclature of medical procedures was provided by the medical informatics department of the establishment (code GFFA009 for “pulmonary lobectomy by thoracoscopy, with or without robot assistance” and GFFA029 for “single or multiple lung segmentectomy”). The data were collected from patients’ medical records and reports (anesthesia and thoracic surgery records).

Exclusion criteria were patients with intraoperative thoracotomy conversion, patients with chronic pain (>3 months), patients with active or past drug addiction (cocaine or heroin), chronic analgesic consumption (>3 months), and age < 18 y/o. A specific identification number for each patient was attributed and the data were collected and analyzed confidentially.

### 2.2. Anesthesia Procedure

The anesthetic procedure for thoracic surgery was protocolized. Prior to surgery, no oral premedication was delivered. All patients were monitored with electrocardiogram, pulse oximetry, and non-invasive blood pressure. The induction was realized with an intravenous (IV) bolus of ketamine (0.25 mg/kg), target-controlled anesthesia (TCI) of remifentanil (Minto pharmacokinetic model using target effect-sited concentration with total body weight for a target between 2 and 4 ng/mL) and propofol (2 to 3 mg/kg). Maintenance of hypnosis and analgesia was realized with sevoflurane with a mixture of air and oxygen (0.8–1 minimum alveolar concentration (MAC) and TCI of remifentanil (target between 2 and 4 ng/mL)). Neuromuscular blockade agents were provided during all interventions (left to the appreciation of the anesthesiologist). Post-operative nausea and vomiting (PONV) were prevented according to Apfel’s score with IV droperidol (1.25 mg) and IV dexamethasone (4 mg) if the patient presented with two or more risk factors.

A double-lumen tube was used for all intubations and the same parameters were used to ventilate the patients (PEP: between 5 to 8 cm H_2_O, tidal volume 6 mL/kg). The adequate position of the double lumen tube was controlled by fiberoptic bronchoscopy after intubation and in the lateral decubitus position. The IV fluid administration was limited to 6 mL/kg/h of crystalloids.

Thirty minutes before the end of the procedure, intraoperative multimodal analgesia was performed, including IV paracetamol (1000 mg), IV nefopam (20 mg), IV ketoprofen (100 mg), and IV morphine (0.15 mg/kg). Following intubation, locoregional chest wall block, ESPB, or PVB was realized. In the ESPB group, ultrasound-guided ESPB was performed in a lateral decubitus position on the thoracoscopic side, out-of-plane technique under aseptic precautions. The tip of the T6 transverse process was identified by progressively downgrading from C7. When the T6 transverse process was in the middle of the image and the pleura were visualized, A 22-gauge block needle was inserted out-plane and guided the middle of the transverse process of T6. The confirmation of the correct location of the needle tip in the fascial deep to the erector spinae muscle was realized by injecting 1 to 2 mL of saline to view the elevation of the erector spinae muscle from the transverse process without distending the muscle. Forty mL of ropivacaine 0.2% (80 mg) was injected into the erector spinae plane. In the PVB group, the ultrasound-guided PVB was performed in a lateral decubitus position on the thoracoscopic side, on the T6 intervertebral level, under ultrasound guidance with 20 mL of ropivacaine 0.2% (40 mg). Following the procedure and after neuromuscular blocking reversal, patients were extubated in the operating room and transferred to the post-anesthesia care unit (PACU). The pain was assessed using a numerical rating scale (NRS), scored from 0 to 10, and was treated if the pain was ≥3/10 in PACU with IV morphine titration. Then, 90 min later, patients returned to the thoracic surgery service if the Aldrete score was adequate. In the service, analgesia prescriptions were protocolized. Patients were administered systematically paracetamol (1 g) every 6 h, ketoprofen (100 mg) every 12 h in the absence of individual contraindications and nefopam (20 mg) every 6 h. If the pain remained and was >6/10, patients also administered morphine sulfate per os, every 6 h (10 mg). If the pain was not managed, the physician could prescribe IV opioids administered through a programmable intravenous patient-controlled analgesia system.

### 2.3. Outcomes Measures

The primary outcome was to compare the level of post-operative pain at rest and at cough at 24 h (H24) between the ESPB group and the PVB group. The pain was assessed using NRS, ranging from 0 to 10.

The secondary outcomes were:

- The level of post-operative pain at rest and cough at the arrival (H0) and the leaving from PACU (H′), and at 6 h (H6). The pain was assessed using NRS, ranging from 0 to 10;
- The post-operative consumption of morphine within the first 24 h. The cumulative consumption of morphine was calculated from PACU until 24 h post-operatively. Results are expressed in IV equivalent;
- Rehabilitation parameters within the first 24 h (chair at 24 h, walking at 24 h, effective coughing at 24 h). Rehabilitation parameters were assessed by a physiotherapist in the surgical department;
- Impact of VATS and RATS on post-operative pain and post-operative consumption of morphine in the ESPB and PVB groups.

Demographic data, surgical procedure, anesthetic, and post-operative data were collected and are available in Supplementary File S1.

### 2.4. Sample Size Calculation

- The sample size was determined from a preliminary retrospective analysis including 20 patients receiving PVB. In these patients, the mean post-operative pain at 24 h was 2 ± 1.8. Considering that a decrease in pain score of 1.2 was clinically relevant with ESPB, a sample size of 48 patients (+15% for non-parametric tests) per group was necessary to show a statistical difference with a power of 90% and a two-sided type I error of 0.05 [12,13,14].

### 2.5. Statistical Analysis

Statistical analyses were performed with R 4.1.0 (the R Foundation for Statistical Computing, http://www.r-project.org (accessed on 2 March 2022)) and Graph Pad v9 (GraphPad Software, San Diego, CA, USA). Using the Shapiro–Wilk test, the Gaussian distribution of the data was explored. Following these explorations, the quantitative variables were compared with a Student t-test if the distribution was normal. A nonparametric Mann–Whitney test was used if the distribution was not normal. For the qualitative variables, depending on the group size, the two samples were compared with a Chi^2^ test or Fisher’s exact test. For continuous measurements, data are presented as the mean ± standard deviation, or median with interquartile ranges [IQR: 25th and 75th percentiles] depending on the distribution of the data. Qualitative parameters are presented as the number of cases n (percentage (%) of patients). As previously described, we used generalized boosted models or a multivariate nonparametric regression technique to estimate the inverse probability weighting (IPTW) in order to control confounding factors in the case of an unknown relationship between the groups (ESPB and PVB) and covariates (age, BMI, ASA score, surgery duration, anesthesia duration, intraoperative total dose of ketamine, of paracetamol, of ketoprofen, of nefopam, and the type of surgery (lobectomy or segmentectomy)) [15,16,17,18,19].

Furthermore, to estimate the regression coefficients (βa, or dependent variable), we used weighted IPTW with generalized linear models (GLM) adjusted for imbalanced covariates. Variables included in the final GLM model were selected using a step-down procedure. Statistical significance was indicated by a value of *p* < 0.05.

## 3. Results

### 3.1. Results

**Figure 1:**
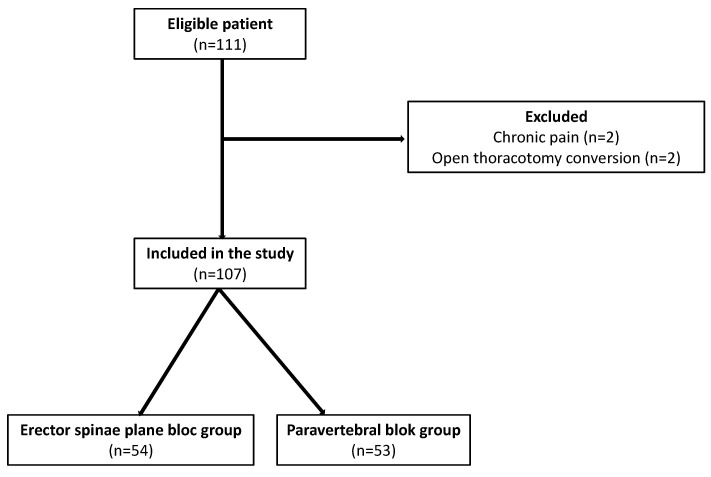
Flowchart of the study.

One hundred and eleven consecutive patients were identified for eligibility between May 2020 and May 2021. Four patients were excluded. There was 54 patients in the ESPB group and 53 in the PVB group (Figure 1). There was no significant difference in the baseline (Supplementary Table S1) and intraoperative characteristics (Supplementary Table S2) between the two groups. Lobectomy was performed for 77 patients (72%) and segmentectomy for 30 patients (28%). The VATS procedure was realized for 50 patients (47%) and RATS procedure for 57 patients (53%).

### 3.2. Post-Operative Pain

The post-operative median pain score at rest and cough was lower in the ESPB group compared to the PVB group at 24 h, respectively, at rest 2 [1; 3.5] vs. 2 [0; 4], *p* = 0.0181 and at cough (4 [3; 6] vs. 5 [4; 6], *p* = 0.0261 (Table 1).

A weighted GLM model analysis confirmed the significant association between ESPB and the decrease of post-operative pain at rest (ESPB −0.80 [−1.50; −0.10], *p* = 0.0255) and at cough at 24 h (ESPB −1.48 [−2.65; −0.31], *p* = 0.0135) (Table 2).

At 6 h, the post-operative median pain score at rest and at cough was lower in the ESPB group compared to the PVB group (respectively at rest 2 (0–3) vs. 3 (1–4), *p* = 0.0099 and at cough 4 (2.7–6) vs. 5 (4–6), *p* = 0.0155) (Table 3). A weighted GLM model analysis confirmed the significant association between ESPB and the decrease of post-operative pain at rest at H6 (ESPB −1 [−1.8; −0,2], *p* = 0.012) and found a tendency at cough at H6 (ESPB −1.3 [−2.6; 0.013], *p* = 0.0524) (Table 2). There was no difference between the 2 groups concerning the post-operative pain at H0 and H′ (Table 3).

### 3.3. Morphine Consumption

There was no difference between the two groups concerning post-operative morphine consumption (Table 2 and Table 3).

### 3.4. Post-Operative Rehabilitation

Rehabilitation evaluated by walking at 24 h was higher in the ESPB group (49 patients (91%)) compared to the PVB group (40 patients (75%)) at 24 h (*p* = 0.04) (Table 3). A weighted GLM model analysis did not confirm this result (*p* = 0.13) (Table 2). Effective cough at D1 was higher in the ESPB group (51 patients (94%)) compared to the PVB group (41 Patients (77%)) (*p* = 0.01) (Table 3). A weighted GLM model analysis confirmed this result (ESPB 0.17 [0.04; 0.29], *p* = 0.008) (Table 2).

### 3.5. Post-Operative Complications

There was no difference between groups concerning the surgical complications (pneumonia *p* = 0.99), post-operative bleeding (*p* = 0.99), persistent air leak (*p* = 0.30) and pneumothorax (*p* = 0.99)), and morphine-related complications (bradypnea (*p* = 0.06) acute urine retention (*p* = 0.99). No patient died during hospitalization and no patient developed acute respiratory distress syndrome.

### 3.6. VATS and RATS Impact

Considering the type of surgery (VATS or RATS), we did not find any difference in the ESPB group for post-operative median pain at 6 h at rest (*p* = 0.84) or cough (*p* = 0.63), at 24 h at rest (*p* = 0.39) or cough (*p* = 0.59), and for post-operative morphine consumption at 24 h (*p* = 0.76). Furthermore, we did not find any difference in the PVB group for post-operative median pain at 6 h at rest (*p* = 0.29) or cough (*p* = 0.77), at 24 h at rest (*p* = 0.80) or cough (*p* = 0.35), and for post-operative morphine consumption at 24 h (*p* = 0.92).

## 4. Discussion

This retrospective study compared a group receiving ESPB and a group receiving PVB to demonstrate the feasibility of ESPB in thoracic surgery, and showed a significant decrease in post-operative pain at 24 h in the ESPB group.

PVB has progressively become the reference technique for analgesia in minimally invasive thoracic surgery compared to the thoracic epidural, and it has shown its superiority to programmable intravenous patient controlled-system analgesia. Nevertheless, the PVB presents risks of complications (failure, vascular puncture, pleural puncture, epidural or intrathecal diffusion, hematoma) even if echo-guided, ultrasound decreased the complication rate. In this context, other blocks, such as the ESPB were explored [20].

The mechanism of action of ESPB is still discussed in the literature with many contradictory studies. The diffusion of the anesthetic solution would be at the thoracolumbar fascia space providing a significant craniocaudal and lateral extension analgesia by blocking the posterior branch of the thoracic spinal nerve, but also at the paravertebral space by blocking the anterior branch of the thoracic spinal nerve and the intercostal nerve. Several cadaveric and imaging studies have supported this hypothesis [6]. The efficiency of SPB compared to the PVB is therefore controversial, but analysis of the literature suggests equivalent efficiency between the two blocks. Indeed, in a recent randomized-controlled non-inferiority study, Zhao et al. showed no difference in the pain score and morphine consumption at 24 h between an ESPB group and PVB group [9]. In contrast to our study, they were two intervertebral injections (T4 and T6) for ESPB and PVB, the patient received systematically post-operatively infusions of non-steroidal anti-inflammatory drugs, patient-controlled IV oxycodone rescue, and there were only 66 patients in this study. In another randomized controlled non-inferiority study, Taketa et al. compared continuous PVB with continuous ESPB and also found no difference in opioid consumption within the first 48 h and pain scores [8]. Another randomized study by Chen et al. comparing post-operative analgesia by ESPB, PVB, or intercostal block, found lower significant median morphine consumption at 24 h in the PVB group (10.5 [9–15] mg) compared to the ESPB group (22.5 [15–25.1] mg). This study also found significantly lower pain scores at 8 h in the PVB group than in the ESPB group. However, this work presented several limits, the ESPB group had only one intervertebral injection and the PVB group had three intervertebral injections. In addition, the patient did not benefit from multimodal analgesia post-operatively, which could explain the difference in median morphine consumption at 24 h between our study and this study [21]. Furthermore, Fang et al., in a monocentric randomized study, found no difference in post-operative pain at rest and cough at 1 h, at 6 h, and 24 h between an ESPB group and PVB group, but found fewer complications in the ESPB group [22]. Compared to our study, this work included patients with open thoracotomy and patients with post-operatively IV patient-controlled analgesia.

The efficacy of the ESPB compared to the PVB remains controversial in the literature, but the analysis suggests a comparable efficacy between the two blocks. However, to our knowledge, our study is the first to compare the efficacy between the two blocks with a single injection of a local anesthetic after VATS or RATS.

The clinical efficacy with the ESPB can be attributed to several factors in our study. First, in the ESPB, the diffusion of the anesthetic solution would be in the thoraco-lumbar fascia allowing for a cranio-caudal and lateral extension by blocking the posterior branch of the spinal nerve, but also the anterior branch of the paravertebral space and the intercostal nerve providing a visceral analgesia [4,6,7,23]. Second, we performed the ESPB with an injected volume of 40 mL and some studies suggest that a volume of at least 30 mL is necessary to obtain a sufficient extension [23]. Third, ESPB is easier to perform than the PVB, and then could compensate for PVB failure rates estimated to range from 4 and 10% [20].

Randomized-controlled superiority trials are needed to legitimize the routine use of the ESPB. However, the efficiency, ease of use, and safety of the ESPB can be attractive as a replacement for the PVB for some clinical situations, and should be kept in mind. Indeed, the PVB can be hazardous when there is a poor visual quality of ultrasound, when the BMI is high, when the patient is taking treatments, such as vitamin K antagonists, direct oral anticoagulants, or heparin, or when there is no chest drain after a surgical procedure (such as a lung wedge resection in ambulatory surgery) or after a block for neuropathic chest pain. The advantages and inconveniences of the ESPB and PVB are summarized in Supplementary Table S3.

This work has limitations. First, although propensity scores reinforced our results in this retrospective study, they did not reveal unobserved differences between the ESPB and PVB groups. Second, this study was a monocentric experience with a restricted number of patients and based on our local practices, and this does not permit us to generalize our results to all thoracic surgery centers widely. Third, the volume of injection of local anesthetics was greater in the ESPB group (40 mL) compared to PVB (20 mL) to obtain a sufficient diffusion, and can explain the difference between groups for post-operative pain. Fourth, the retrospective nature of our study did not allow us to compare the dermatomal distribution of the sensory blockade between the groups. Finally, we were not able to evaluate the impact of ESPB on chronic pain at 3 months, a topic of particular interest in thoracic surgery.

## 5. Conclusions

ESPB was associated with lower pain scores at 24 h compared to PVB. However, the clinical relevance of the results is less clear. Although, this study suggests that ESPB is an acceptable and safe alternative compared to PVB, ESPB should be kept in mind by the anesthesiologist when the PVB is difficult or hazardous. A regional procedure, such as ESPB or PVB should be used whenever possible in VATS or RATS for lung resection.

## Figures and Tables

**Table 1:** Post-operative pain at rest and cough at 24 h.

	Total (n = 107)	ESPB Group (n = 54)	PVB Group (n = 53)	*p*
Primary Outcome				
Post-operative pain at rest at 24 h (numerical rating scale from 0 to 10)	2 [0; 3]	2 [1; 3.5]	2 [0; 4]	0.0181
Post-operative pain at cough at 24 h (numerical rating scale from 0 to 10)	4 [3; 6]	4 [3; 6]	5 [4; 6]	0.0261

Data are expressed as median [25–75th percentile].

**Table 2:** Propensity score analysis.

Endpoints							
	Level of Post-Operative Pain at 24 h at Rest	Level of Post-Operative Pain at 24 h at Cough	Level of Post-Operative Pain at 6 h at Rest	Level of Post-Operative Pain at 6 h at Cough	Cumulative Consumption of Morphine at 24 h (mg)	Rehabilitation: Walking at 24 h	Rehabilitation Effective Cough at Day 1
Included covariate	Change or Adjusted ß_a_ 95 IC *p*-value of double-adjustment in the propensity score matching analysis						
Erector spinae plane block Main predictor	−0.80 [−1.50; −0.10] *p* = 0.0255	−1.48 [−2.65; −0.31] *p* = 0.0135	−1 [−1.8; −0,2] *p* = 0.012	−1.3 [−2.6; 0.013] *p* = 0.0524	−0.23 [−4.54; 4.07] *p* = 0.91	0.13 [−0.01; 0.27] *p* = 0.13	0.17 [0.04; 0.29] *p* = 0.008

Results are expressed in per os equivalent for cumulative morphine consumption. The pain was measured using a numerical rating scale, range 0 to 10.

**Table 3:** Post-operative pain, morphine consumption and post-operative rehabilitation.

	Gobal Population (n = 107)	ESPB Group (n = 54)	PVB Group (n = 53)	*p*-Value
**Pain and PONV in PACU**				
Time in PACU (min)	85 (65–115)	80 (60–120)	90 (67.50–112.50)	0.42
Level of post-operative pain at rest at arrival in PACU	0 (0–3)	0 (0–3)	0 (0–4)	0.99
Level of post-operative pain at cough when arrival in PACU	3 (0–5)	2 (0–5)	3 (0–5)	0.80
Level of post-operative pain at rest when leaving PACU	2 (0–3)	2 (0–3)	2 (0–3)	0.42
Level of post-operative pain at cough when leaving PACU	3 (2–4)	3 (1–4)	3 (2–5)	0.09
PONV	11 (10)	6 (11)	5 (9)	0.99
**Pain at 6 h (NRS, range 0 to 10)**				
Pain at rest at H6	2 (0–4)	2 (0–3)	3 (1–4)	0.0099
Pain at cough at H6	4 (3–6)	4 (2.75–6)	5 (4–6)	0.0155
**Non-morphine analgesics at 24 h**				
Paracetamol	105 (98)	53 (98)	52 (98)	0.99
Nefopam	74 (69)	36 (67)	38 (72)	0.57
Ketoprofen	74 (69)	34 (63)	40 (75)	0.16
**Cumulative morphine consumption at 24 h (from PACU)**				
Cumulative morphine consumption at 24 h (mg)	6.67 (3.33–10)	6.33 (2.50–9.17)	6.67 (3.33–10.20)	0.19
**Rehabilitation**				
Chair at 24 h	102 (95)	53 (98)	49 (92)	0.20
Walking at 24 h	89 (83)	49 (91)	40 (75)	0.04
Effective coughing	92 (86)	51 (94)	41 (77)	0.01
Acute urine retention	3 (3)	2 (4)	1 (2)	0.99
Bradypnea	4 (4)	0 (0)	4 (8)	0.06
Chest drainage time (day)	3 (2–6)	3 (2–6)	3 (2–5)	0.82
Time of hospital stay (day)	4 (3–6)	4 (3–6)	3 (2–6)	0.25
**Complications**				
Persistent air leak	16 (15)	10 (19)	6 (11)	0.30
Pneumonia	6 (6)	3 (6)	3 (6)	0.99
Pneumothorax	5 (5)	2 (4)	3 (6)	0.66
Post-operative bleeding	1 (2)	1 (2)	0 (0)	0.99

Data for the normal distribution are expressed as the mean ± SD. For the non-normal distribution, data are expressed as median [25–75th percentile]. For qualitative parameters, data are presented as n (% of patients). Abbreviations: NRS, numerical rating scale; PACU, post-anesthesia care unit; PONV, post-operative nausea and vomiting. For morphine consumption, results are expressed in per os equivalent.

## Data Availability

The data underlying this article will be shared upon reasonable request to the corresponding author.

## Supplementary Materials

The following supporting information can be downloaded at: https://www.mdpi.com/article/10.3390/cancers15082306/s1, File S1: Demographic data, surgical procedure, anesthetic, and post-operative data collected; Table S1: Baseline Characteristics; Table S2: Patients’ intraoperative characteristics; Table S3: Advantages and inconveniences of the PVB and ESPB.
