# Supplementary material for: Erector Spinae Plane Block versus Paravertebral Block after Thoracic Surgery for Lung Cancer: A Propensity Score Study
Source: Cancers (Basel). 2023 Apr 14;15(8):2306. doi: 10.3390/cancers15082306 (PMC10137003; doi:10.3390/cancers15082306)
Supplement: Supplementary file 1 [file cancers-15-02306-s001.zip › Supplementary File S1.pdf]

Supplementary File S1: Demographic data, surgical procedure, anesthetic and post-operative data collected

Demographic data: gender, age, ASA score, body mass index (BMI) comorbidities (chronic analgesic consumption, neoadjuvant chemotherapy, chronic obstructive pulmonary disease, diabetes, arterial hypertension, cardiac insufficiency, renal insufficiency (clearance <30 mL/min), hepatic Insufficiency, coronary artery disease). **Anesthetic data:** the total dose of ketamine, the total dose of remifentanil, the dose of intraoperative morphine. The dexamethasone, paracetamol, nefopam and ketoprofen use. **Surgical procedure data**: duration and type of intervention (lobectomy or segmentectomy, video-assisted thoracoscopy, or robot-assisted thoracoscopy). **Postoperative data**: time in Post-Anesthesia Care Unit (PACU), level of postoperative pain at rest at arrival in PACU, level of postoperative pain at cough when arrival in PACU, Level of postoperative pain at rest when leaving PACU, Level of postoperative pain at cough when leaving PACU, the incidence of postoperative nausea and vomiting (PONV) in PACU. Non-morphine analgesics at 24-hr (paracetamol, nefopam, ketoprofen), morphine-related complications (acute urine retention, bradypnea, chest drainage time (day), length of hospital stay (day), and other complications (persistent air leak, pneumonia, pneumothorax, post-operative bleeding).
