# Supplementary material for: Erector Spinae Plane Block versus Paravertebral Block after Thoracic Surgery for Lung Cancer: A Propensity Score Study
Source: Cancers (Basel). 2023 Apr 14;15(8):2306. doi: 10.3390/cancers15082306 (PMC10137003; doi:10.3390/cancers15082306)
Supplement: Supplementary file 1 [file cancers-15-02306-s001.zip › Supplementary Table S1.pdf]

Table S1. Baseline characteristics.

	Global population (n=107) (%)	ESPB group (n=54) (%)	BPV group (n=53) (%)	p-value
**Demographic characteristics**				
Age (yr, range)	65 [59-70]	66 [60-71]	63 [57-69]	0.08
Sex				
Male	66 (62)	36 (67)	30 (57)	0.28
Female	41 (38)	18 (33)	23 (43)	0.28
ASA 1	11 (10)	3 (6)	8 (15)	0.12
ASA 2	82 (77)	41 (76)	41 (77)	0.86
ASA 3	14 (13)	10 (18)	4 (8)	0.15
Body mass index (kg/m^2)	26.05 ±4.82	26.89 ±5.26	25.19 ±4.21	0.06
**Comorbidities**				
Chronic analgesic consumption	8 (7)	3 (6)	5 (9)	0.48
Neoadjuvant chemotherapy	5 (5)	3 (6)	2 (4)	0.90
COPD	28 (26)	18 (33)	10 (19)	0.08
Diabetes	11 (10)	7 (13)	4 (8)	0.53
Arterial hypertension	40 (37)	22 (41)	18 (34)	0.47
Cardiac insufficiency	6 (6)	4 (7)	2 (4)	0.68
Renal insufficiency (clearance <30 mL/min)	9 (8)	6 (11)	3 (5)	0.49
Hepatic Insufficiency	0 (0)	0 (0)	0 (0)	NA
Coronary artery disease	9 (8)	5 (9)	4 (8)	0.99

Data for normal distribution are expressed as mean ± SD. For non-normal distribution, data are expressed as median [25-75th percentile]. For qualitative parameters, data are presented as n (% of patients).

Abbreviations: ASA, American Society of Anaesthesiologists; COPD, Chronic obstructive pulmonary disease; ESPB group, Erector spinae plane block group; PVB group, Paravertebral block group.

NA, statistical tests are not applicable due to the number of patients.
