# Supplementary material for: Erector Spinae Plane Block versus Paravertebral Block after Thoracic Surgery for Lung Cancer: A Propensity Score Study
Source: Cancers (Basel). 2023 Apr 14;15(8):2306. doi: 10.3390/cancers15082306 (PMC10137003; doi:10.3390/cancers15082306)
Supplement: Supplementary file 1 [file cancers-15-02306-s001.zip › Supplementary Table S2.pdf]

Supplementary Table S2. Patients' intraoperative characteristics.

	Global population (n=107) (%)	ESPB group (n=54) (%)	BPV group (n=53) (%)	p-value
**Surgery**				
Lobectomy	77 (72%)	36 (67%)	41 (77%)	0.22
Segmentectomy	30 (28%)	18 (33%)	12 (23%)	0.22
VATS	50 (47%)	27 (50%)	23 (43%)	0.49
RATS	57 (53%)	27 (50%)	30 (57%)	0.49
Surgery duration (min)	141.90 ±41.98	134.60 ±43.50	149.20 ±39.43	0.07
**Anesthesia and analgesia during surgery**				
Total dose of ketamine (mg)	46 [35-50]	40 [30-50]	50 [40-50]	0.26
Total dose of remifentanil (µg)	768 [628-1150]	752 [598-946]	851 [641-1240]	0.07
Intraoperative morphine (mg)	10 [8-10]	10 [8-10]	9 [8-10]	0.15
Intraoperative dexamethasone	103 (96)	51 (94)	52 (98)	0.62
Intraoperative paracetamol	107 (100)	54 (100)	53 (100)	0.99
Intraoperative nefopam	102 (95)	53 (98)	59 (92)	0.2
Intraoperative ketoprofen	90 (84)	46 (85)	44 (83)	0.76

Data for normal distribution are expressed as mean ± SD. For non-normal distribution, data are expressed as median [25-75th percentile]. For qualitative parameters, data are presented as n (% of patients).
Abbreviations: VATS, Video-assisted thoracoscopy; RATS, Robot-assisted thoracoscopy.
