# Supplementary material for: Erector Spinae Plane Block versus Paravertebral Block after Thoracic Surgery for Lung Cancer: A Propensity Score Study
Source: Cancers (Basel). 2023 Apr 14;15(8):2306. doi: 10.3390/cancers15082306 (PMC10137003; doi:10.3390/cancers15082306)
Supplement: Supplementary file 1 [file cancers-15-02306-s001.zip › Supplementary Table S3.pdf]

Supplementary Table S3. Advantages and inconveniences of the PVB and ESPB.

	Advantages	Inconveniences
**Paravertebral block (PVB)**	- Important bibliography reporting similar efficacy between continuous PVB and thoracic epidural analgesia (EA) for postoperative pain and morphine consumption after videothoracoscopy<br>- Fewer side effects than thoracic EA (hypotension, nausea)<br>- Possible continuous analgesia with catheter<br>- Ipsilateral to surgery<br>- Can be performed on a patient under general anesthesia	- Frequent complications (pneumothorax, intravascular injections, intrathecal injections).<br>- Contraindication under curative anticoagulation (risk of spinal epidural hematoma)<br>- Challenging block requiring expertise and experience in locoregional anesthesia
**Erector spinae plane block (ESPB)**	- Superficial compared to PVB: infrequent complications (space away from the pleura, large vessels, and the epidural and intrathecal space)<br>- Interesting block for analgesia in ambulatory surgery and locoregional anesthesia for chronic pain<br>- Block can be performed under curative anticoagulation<br>- Possible continuous analgesia with catheter<br>- Ipsilateral to surgery	- There is a lack of evidence for this block for routine use, but recent studies suggest a similar efficacy to PVB regarding postoperative pain and morphine consumption

	- Can be performed on a patient under general anesthesia	

Abbreviations; EA, epidural analgesia; ESPB, erector spinae plane block; PVB, Paravertebral block.
